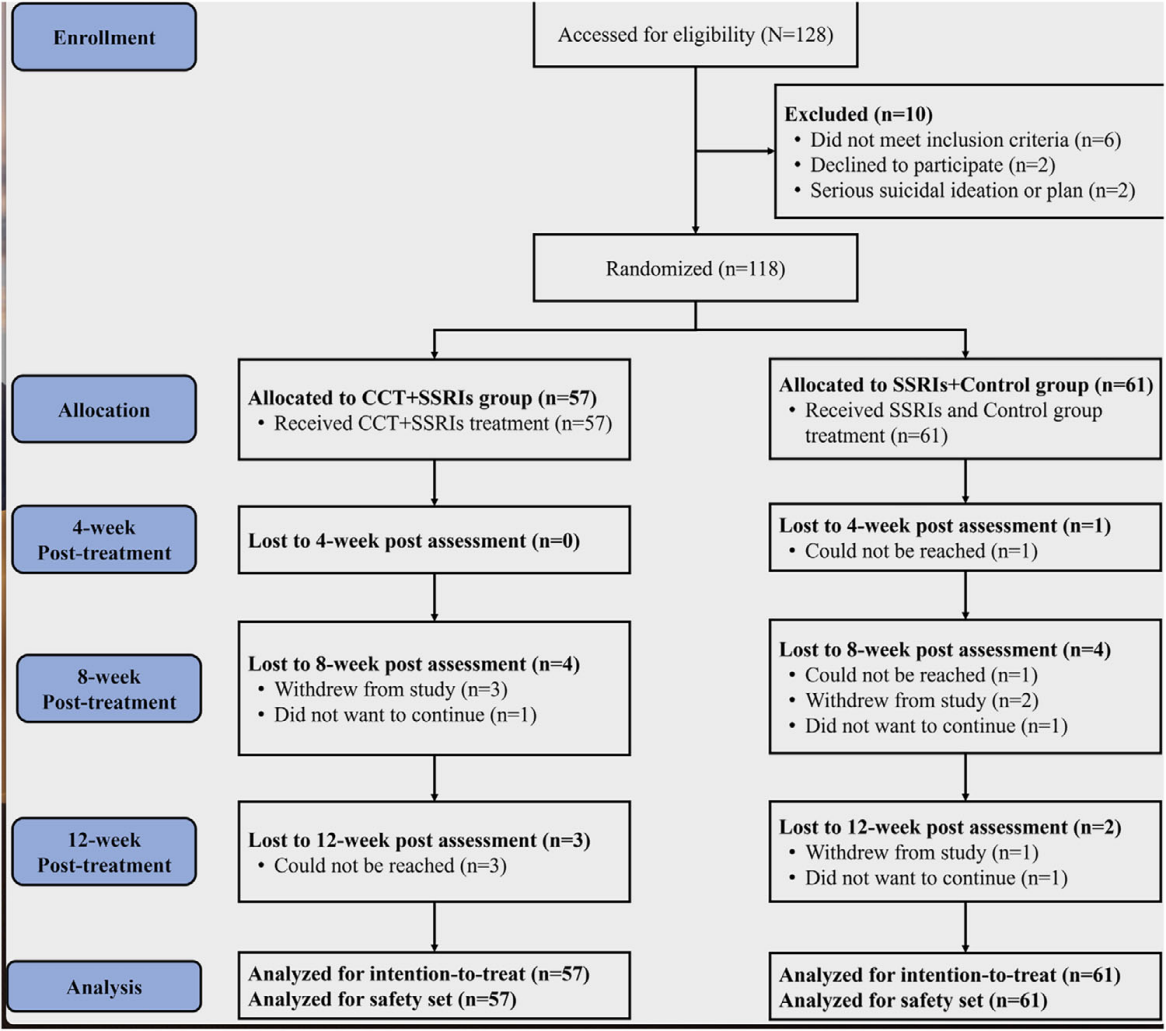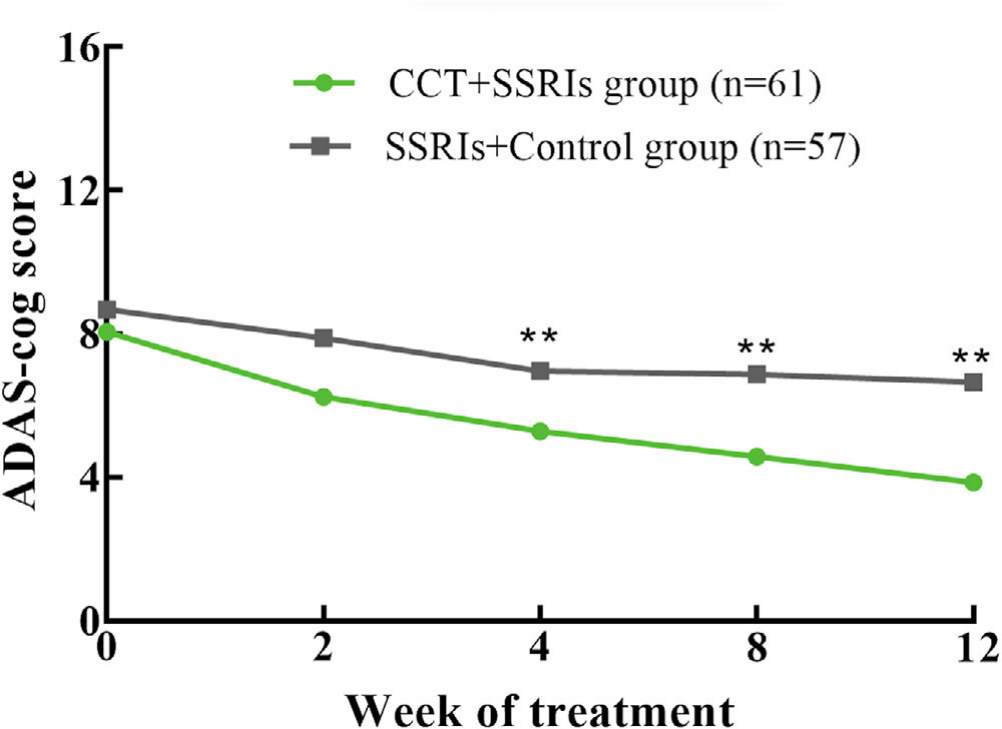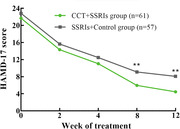# Efficacy and safety of computerized cognitive training combined with SSRIs for treating cognitive impairment among patients with late‐life depression: A 12‐week, randomized controlled study

**DOI:** 10.1002/alz.085838

**Published:** 2025-01-03

**Authors:** Xiao Wang, Qinge Zhang

**Affiliations:** ^1^ Beijing anding hospital, Beijing, Beijing China

## Abstract

**背景:Background:**

This randomized, open‐label study examined the therapeutic effects of computerized cognitive training (CCT) combined with selective serotonin reuptake inhibitors (SSRIs) on cognitive impairment among patients with late‐life depression (LLD).

**方法: Method:**

Study data were collected from May 5, 2021, to April 21, 2023. Outpatients who met diagnostic criteria for major depressive disorder according to the fifth revision of the Diagnostic and Statistical Manual of Mental Disorders (DSM‐5) criteria (HAMD‐17) ≥ 18 and a total score on the MOCA) <26 were randomly assigned to receive up to 12 weeks of CCT and SSRIs treatment (n = 57) or SSRIs and Control treatment (n = 61). The primary outcome was the change in Alzheimer’s Disease Assessment Scale‐Cognitive Subscale (ADAS‐Cog) scores from baseline to week 12 between the two groups. Mixed model repeated measures (MMRM) analysis was performed on modified intention‐to‐treat (mITT) and completer populations.

**结果: Result:**

The full analysis set (FAS) included 118 patients (CCT and SSRIs group, n = 57; SSRIs and Control group, n = 61). Over the 12‐week study period, the reduction in the ADAS‐cog total score was significant (P < 0.001) in both groups, while MMRM analysis revealed a significantly greater reduction in cognitive function (ADAS‐cog total scores) from baseline to posttreatment in the CCT and SSRIs group than in the SSRI and Control group [(F (1,115) = 13.65, least‐squares mean difference [95% CI]: −2.77 [−3.73, −1.81], p<0.001)]. There were significantly greater improvements in depression symptoms (measured by the HAMD‐17) in the CCT and SSRIs group than in the control group [MMRM, estimated mean difference (SE) between groups −3.59 [−5.02, −2.15], p < 0.001]. The least‐squares mean changes in the HAMA scores and NPI scores between baseline and week 8 were greater in the CCT and SSRIs group than in the control group (all P < 0.05). The most frequent adverse events (AEs) in both groups were dry mouth, somnolence, and constipation. There was no significant difference in the incidence of adverse events between the two groups.

**结论:Conclusion:**

CCT combined with SSRIs was efficacious and well tolerated in LLD patients with cognitive impairment.